# Primary Sternal Osteomyelitis Caused by Methicillin-Sensitive Staphylococcus aureus: A Diagnosis Rare in Healthy Adults

**DOI:** 10.7759/cureus.16080

**Published:** 2021-07-01

**Authors:** Tiago Araújo, Monika Dvorakova, Leonor Gama, Yulia Shigaeva, Teresa Bernardo

**Affiliations:** 1 Internal Medicine, Unidade Local de Saúde do Litoral Alentejano, Santiago do Cacém, PRT

**Keywords:** sternal osteomyelitis, primary, staphylococcus aureus, bloodstream infection, immuno-competent host

## Abstract

Primary sternal osteomyelitis (PSO) is a rare clinical entity, and usually, it is associated with predisposing factors such as intravenous drug use, diabetes mellitus, or human deficiency virus infection. In an otherwise healthy adult, it becomes an even rarer entity. Early diagnosis and treatment minimize associated morbidity, like the need for surgical debridement, longer courses of medication, and length of in-hospital stay.

We describe the case of a 54-year-old man without any predisposing risk factors for PSO, who presented with chest pain, erythema, tenderness, and warmth at the right parasternal region. A non-enhanced thoracic tomography showed a 33 mm suspicious pulmonary nodule and no signs of sternum abnormalities. To better evaluate this finding, a positron emission tomography with fluorine-18 fluorodeoxyglucose was performed, showing abnormal uptake of the radionuclide at the sternomanubrial synchondrosis and no abnormal uptake at the lung parenchyma. The presence of *Staphylococcus aureus* in blood cultures, in conjunction with these results, supported the diagnosis of PSO. The patient completed six weeks of microbiologically oriented antibacterial therapy with complete recovery.

## Introduction

The most common type of sternal osteomyelitis is named secondary sternal osteomyelitis and it is most commonly associated with sternotomy for cardiac surgery, chest trauma, subclavian vein catheterization or closed cardiopulmonary resuscitation [1]. Primary sternal osteomyelitis (PSO) occurs by hematogenous seeding of the sternum in the setting of bacteremia. The most common risk factors for this condition are intravenous drug use, diabetes mellitus, and human immunodeficiency virus (HIV) infection [2]. Treatment usually consists of antimicrobial therapy, but in more severe cases surgery might be needed [3]. In healthy individuals with no risk factors for PSO, the diagnosis and treatment may be delayed excessively, increasing morbidity and mortality. This article was previously presented as a poster at the 2019 Portuguese Society of Internal Medicine Annual Congress, on May 24, 2021.

## Case presentation

A 54-year-old man with a personal history of hypertension and hypercholesterolemia presented to the emergency department (ED) with a 24-hour history of stabbing chest pain at the right sternomanubrial angle region. The pain did not radiate to other locations and was made worse by touch, deep respiratory breaths, and right arm movements. There was no history of fever, nausea, dizziness, shortness of breath, other respiratory symptoms. Similarly, a history of chest trauma or surgery was also absent.

On physical examination, the patient appeared to be in a good general condition, was hemodynamically stable, afebrile, without abnormal cardiac, pulmonary, or abdominal findings. The right parasternal area was tender, but without swelling, erythema, warmth, or any other inflammatory sign. A chest x-ray was performed and was within normal limits. Electrocardiogram (ECG) showed sinus rhythm with no signs of myocardial ischemia. The available laboratory data showed Troponin-I of 0.01 ng/mL (normal range <00.5 ng/mL), total creatine kinase (Total-CK) of 118 U/L (normal range 49-397 U/L), creatine kinase myocardial band (CK-MB) of 1.1 ng/mL (normal range 0.6-6.3 ng/mL) and slightly elevated lactate dehydrogenase of 670 U/L (normal range 266-500 U/L). The patient was discharged with a prescription of non-steroidal anti-inflammatory drugs (NSAIDs) and muscle relaxants for suspected costochondritis of undetermined cause.

**Figure 1:**
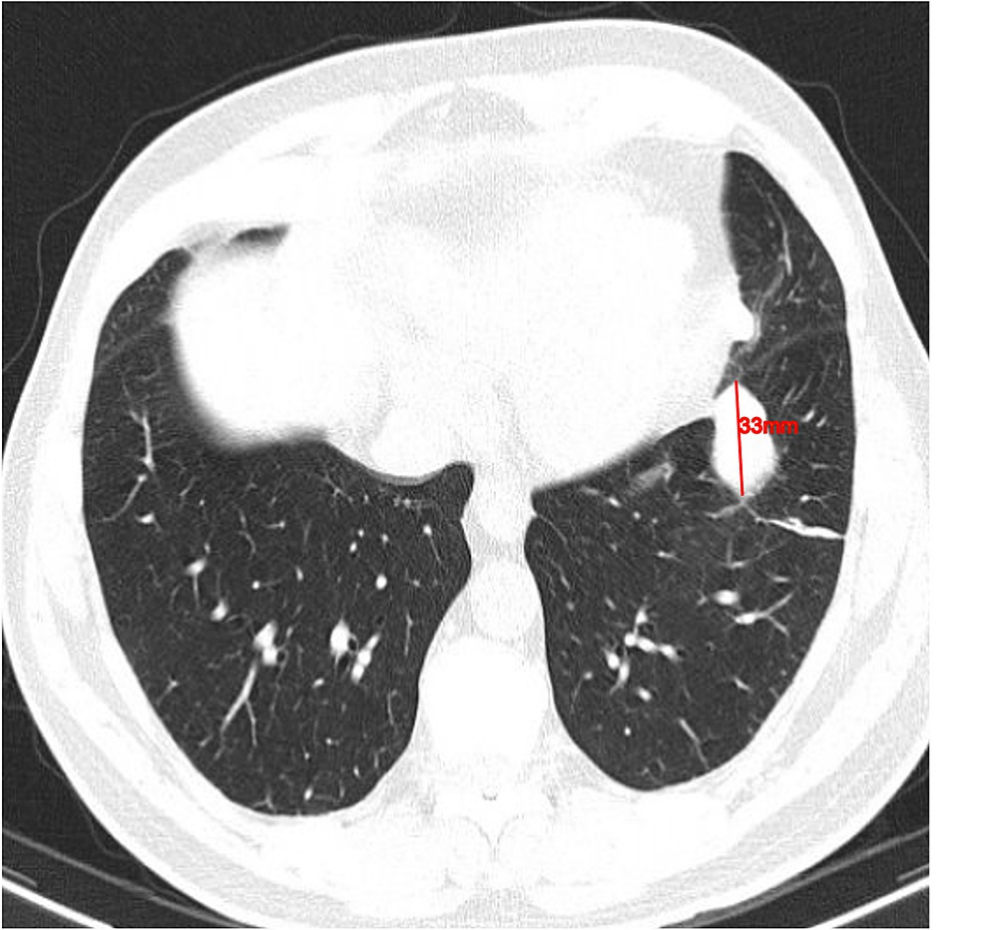
Non-enhanced computed tomography Juxta-pleural thickening, in a nodular shape, of 33 mm longitudinal diameter in the left inferior lobe.

He returned 14 days later, maintaining complaints of a stabbing chest pain at the same location and with the same characteristics, however, associated with new-onset redness and swelling of the right parasternal region. He denied fever or other symptoms. On physical examination he was hemodynamically stable, his blood pressure was 142/82 mmHg, heart rate of 58 beats/minute, and body temperature of 35.6°C. Moderate swelling and warmth of the right parasternal area were present with aggravated tenderness, but no other inflammatory signs. New ECG and chest x-ray showed no changes from the previous ones. Laboratory data showed leukocytosis (13.7 x10^3^ cells/µL) with neutrophilia (10.4 x10^3^ cells/µL); elevated Total-CK of 540 U/L (normal range 49-397 U/L); elevated C-reactive protein of 9.7 mg/dL (normal range <1.0 mg/dL). A non-enhanced thoracic computerized tomography (CT) showed a juxta-pleural thickening with a nodular shape and irregular margins of 33 mm longitudinal diameter on the left inferior lobe but no suspicious findings on the sternum (Figure 1).

**Figure 2:**
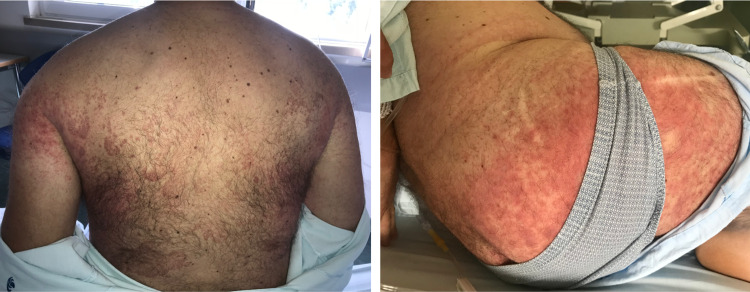
Staphylococcus aureus folliculitis Multiple infected hair follicles with edematous papules and pustules, associated with erythema and warmth.

The patient was admitted to continue the etiologic study of the pulmonary nodule. We suspected that the patient had idiopathic Tietze Syndrome, but since he was afebrile and hemodynamically stable, NSAID therapy was restarted and no antimicrobial therapy was initiated. A urine culture and two sets of blood cultures were collected. During the next 48 hours, the patient showed no signs of improvement in chest pain or the associated regional inflammation. After displaying a lack of improvement for 48 hours, the patient developed a generalized skin rash with erythematous papules and pustules (Figure 2).

At the same time, a significant rise in C-reactive protein levels occurred (from 9.7 mg/dL to 19.2 mg/dL), associated with an erythrocyte sedimentation rate (ESR) of 80 mm/first hour. Besides, the blood cultures performed on admission revealed methicillin-susceptible *Staphylococcus aureus* (MSSA). The urine culture was sterile. The patient was immediately started on antimicrobial therapy with intravenous flucloxacillin (12 grams a day). Although the patient met only one major clinical criteria among the Duke criteria for the diagnosis of infective endocarditis (positive blood cultures featuring an infective agent that commonly causes infective endocarditis), a transthoracic echocardiogram was performed. It showed an absence of signs indicative of endocardic involvement associated with the aforementioned bacteremia. Antibody testing for B and C hepatitis viruses and Human Immunodeficiency virus were negative and the patient denied the use of intravenous drugs. For additional evaluation of the pulmonary nodule reported in the initial thoracic CT, a flexible bronchofibroscopy was performed, which showed no structural abnormalities and anatomopathological examination of the bronchoalveolar lavage was negative for malignant cells. Mycologic and mycobacteriology examinations of the bronchoalveolar lavage were also negative, but the bacterial culture was positive for MSSA.

**Figure 3:**
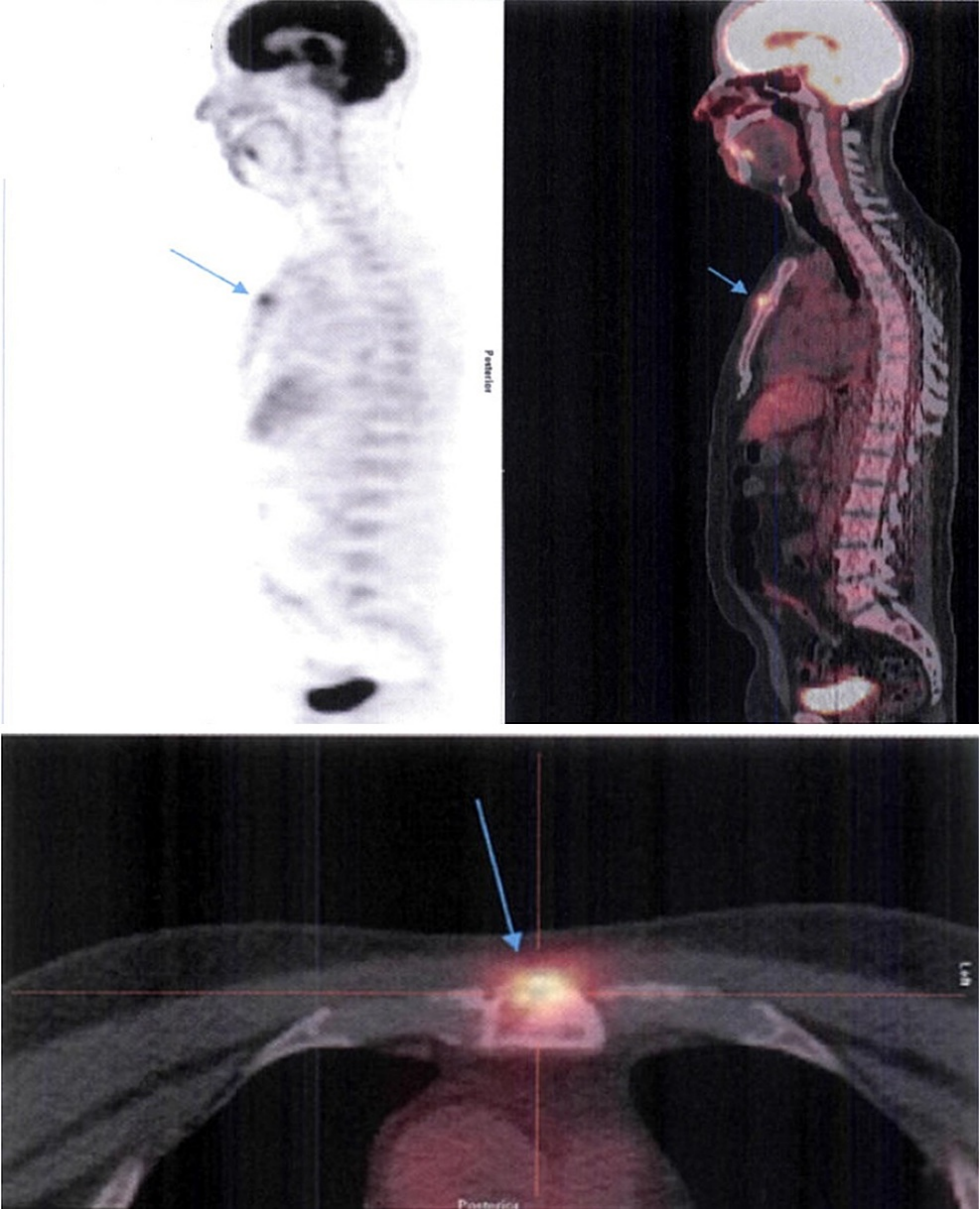
Fluorine-18 fluorodeoxyglucose positron emission tomography (F-18-FGD PET) performed after 15 days of antimicrobial therapy Juxta-centimetric lesion showing increased uptake of F-18-FGD by the small osteolytic area at the sternomanubrial synchondrosis (blue arrows).

Throughout the first ten days of antimicrobial therapy, the patient showed steady improvement of the chest pain; the swelling and tenderness and the skin rash completely subsided. These findings were corroborated by the normalization of the previously elevated inflammatory markers. He was discharged on the fourteenth day after admission (tenth day of intravenous flucloxacillin), with instructions to complete 21 days of antimicrobial therapy with oral flucloxacillin. Five days after discharge a fluorine-18 fluorodeoxyglucose (F-18-FGD) positron emission tomography (FDG-PET) scan was performed to better evaluate the pulmonary nodule. It showed a juxta-centimetric lesion characterized by increased uptake of F-18-FGD at the sternomanubrial synchondrosis, which was highly suggestive of osteomyelitis (Figure 3). There were no suspicious lesions in the pulmonary parenchyma; the nodule seen in the thoracic CT was a juxta-diaphragmatic thickening with no anomalous uptake of F-18-FDG.

Following confirmation of the PSO diagnosis, the patient was instructed to extend oral antimicrobial therapy, achieving a total length of six weeks. After that period, he was reexamined in the outpatient clinic. His chest pain, tenderness, and swelling had completely subsided. A new thoracic CT was performed 20 days after completion of the antimicrobial therapy, which revealed no sternal sequelae.

## Discussion

Osteomyelitis is a predominantly bacterial infection of the bone that causes its destruction. It can develop through three different mechanisms: hematogenous dissemination in the setting of bacteremia; contiguous spread of infection from adjacent soft tissues and joints, such as in the case of diabetic foot wounds; or through direct penetration of a microorganism after trauma or surgery [4]. There are many risk factors associated with hematogenous osteomyelitis, the most common being intravenous drug use, diabetes mellitus, immunodeficiency, subclavian vein catheterization, or liver cirrhosis [5-6]. Among adults, the most frequent form of hematogenous osteomyelitis is vertebral osteomyelitis [5]. In contrast, PSO is an extremely rare condition, being responsible for only 0.3% of all cases of osteomyelitis [6].

In the adult population, hematogenous osteomyelitis is usually caused by a single microorganism, the most common being *Staphylococcus aureus* (susceptible or resistant to methicillin) [7]. However, depending on the pathophysiological type of PSO and epidemiologic factors, many other pathogens can lead to osteomyelitis by hematogenous dissemination. Among intravenous drug users, gram-negative rods as *Pseudomonas aeruginosa* and Gram-positive cocci like staphylococci are common pathogens [8]. In patients with HIV or other immunodeficiency disorders other frequently isolated microorganisms are *Bartonella henselae* and *Bartonella quintana*, *Aspergillus *spp., *Candida albicans*, and *Mycobacteria *spp. [9]. In sickle-cell disease, *Salmonella *spp. and *Streptococcus pneumoniae* are also frequent pathogens [9]. *Staphylococcus aureus* has a set of virulence factors that increase its pathogenicity towards osteomyelitis: first, bacterial adhesins promote attachment to extracellular matrix; secondly, toxins, capsular polysaccharides, and protein A promote evasion from host defenses; and finally, exotoxins and various hydrolases promote invasion and tissue penetration [9]. Osteomyelitis related to methicillin-resistant* Staphylococcus aureus* (MRSA) tends to be more symptomatic, with greater morbidity, when compared to MSSA-associated osteomyelitis [10].

PSO commonly presents as chest pain, tenderness, swelling, warmth, and erythema of the anterior thoracic wall, associated with fever and chills [6]. When our patient presented to the ED for the first time, his main complaint was chest pain. There is a need to contemplate numerous cardiac and non-cardiac causes for chest pain when performing its differential diagnosis. A detailed anamnesis is extremely important to differentiate which of them is more probable and it must be accompanied by key complementary exams. Musculoskeletal causes for chest pain, like costochondritis or Tietze Syndrome, mimic much of the symptoms of PSO. In a patient with fever, cardiac murmur, or leukocytosis, infectious causes for chest pain must be considered. It is also very important to keep in mind, that patients with immunodeficiency disorders may not be able to mount a fever, even in advanced stages of osteomyelitis, requiring an even higher index of suspicion when presenting with persistent musculoskeletal pain. Since PSO is a rare entity, it might be mistakenly confused with other causes of chest pain, delaying its diagnosis and the start of adequate treatment. When our patient visited the ED, he denied ever having a fever; he had only mildly elevated inflammatory markers and thus was erroneously diagnosed with costochondritis and later with Tietze Syndrome when the swelling, erythema, and warmth developed.

Hematogenous osteomyelitis should be considered when a patient presents new or worsening musculoskeletal pain, especially in the setting of fever and/or bacteremia [5]. The diagnosis of PSO is based on a combination of clinical suspicion, laboratory findings, microbiological exams, radiologic imaging, and sometimes, even bone biopsy. Elevated C-reactive protein, erythrocyte sedimentation rate, and white blood cell count are usually present. Blood cultures are positive in 25-50% of patients with hematogenous osteomyelitis [4]. The gold standard for the diagnosis of osteomyelitis is bone biopsy with histopathologic examination and culture of bone samples [11]. However, in non-vertebral osteomyelitis the diagnostic yield of bone biopsy is low (34-68%) [12]. When blood cultures and radiologic findings are highly suggestive of osteomyelitis, bone biopsy may be postponed, and empiric antimicrobial therapy started [3]. The radiologic imaging techniques include plain radiography, CT scan, magnetic resonance imaging (MRI), and nuclear medicine studies. Plain radiography has low sensitivity and specificity for diagnosing acute osteomyelitis, mainly because nearly 80% of patients will have a normal x-ray in the first two weeks of disease onset [13]. MRI is the gold standard in imaging for osteomyelitis because of its anatomical detail and high sensitivity to early disease (82-100%) [13, 14]. Gadolinium-enhanced MRI may be useful to differentiate an abscess from a phlegmon, or in cases where an epiphyseal infection is suspected [13]. CT is less expensive than MRI, is widely available, and has better bone resolution [13]. Nevertheless, its limitation arises from its poor soft tissue resolution; since it is unable to demonstrate bone marrow edema, a normal CT cannot exclude osteomyelitis [13]. Nuclear medicine studies demonstrate sites of abnormal bone metabolism, manifested by areas of increased uptake of the injected radionuclide. These studies, like FDG-PET, triple-phase bone scans with technetium-99m-labelled methyl diphosphonate, gallium scans, or white cell scans, have very high sensitivity in detecting osteomyelitis and grant the advantage of allowing imaging of the entire body, to look for multiple sites of infection [13]. However, these studies lack specificity and anatomical localization [13]. If a site of abnormal radionuclide uptake is found, additional confirmation with MRI or bone biopsy is needed to establish the diagnosis of osteomyelitis [13]. Our patient underwent plain chest radiography and chest CT on admission, neither of which showed any changes in the sternum. Furthermore, the finding of a suspicious pulmonary nodule diverted the medical team’s attention from his main complaint; osteomyelitis was not suspected, despite the patient having *Staphylococcus aureus* bacteremia, until the FDG-PET was performed. At that moment, the patient had already completed 15 days of antimicrobial therapy, and his chest complaints had subsided almost entirely, so it was decided not to perform MRI or bone biopsy. In this patient, the absence of any risk factors for hematogenous osteomyelitis, combined with lack of radiologic changes in the sternum, were also contributing factors for the delay in diagnosis. 

The mainstay treatment for nonvertebral hematogenous osteomyelitis is parenteral antimicrobial therapy directed against the causative pathogen. Surgical debridement might be needed in patients with subperiosteal collection or abscess, necrotic bone, and/or simultaneous joint infection [3]. Before the identification of the responsible microorganism in blood cultures or bone biopsy, empiric antimicrobial therapy for hematogenous osteomyelitis should include coverage against *Staphylococcus aureus*, with beta-lactams like flucloxacillin, nafcillin, or oxacillin [4, 15]. Furthermore, if there is a risk of MRSA infection, vancomycin is the agent of choice [4, 15]. Coverage for aerobic Gram-negative bacilli should also be included in empiric antimicrobial therapy for hematogenous osteomyelitis with a third- or fourth-generation cephalosporin [3]. The duration of antimicrobial therapy depends on whether surgical debridement was performed or not, but at least six weeks should be completed [4]. PSO might be effectively treated with antibiotics alone; however, surgical debridement and drainage must be considered always [16]. When extensive surgical debridement is needed it should be followed by reconstructive procedures like bone grafting or muscle flap rotation [3, 17]. Hyperbaric oxygen therapy might improve wound healing diminishing the need for reconstructive procedures [17].

## Conclusions

PSO is a rare entity, with a clinical presentation that may lead to it being mistakenly confused with other causes of musculoskeletal pain, particularly in a patient without risk factors. This diagnostic challenge also occurred in our case. In a patient presenting with fever and chest pain, infectious causes must be considered, and a detailed anamnesis and physical examination must be acquired to effectively distinguish cardiogenic and non-cardiogenic causes. Early diagnosis and treatment are important to limit associated morbidity.
